# Lung Middle Lobe Laceration Needing Lobectomy as Complication of Nuss Bar Removal

**DOI:** 10.1155/2018/8965641

**Published:** 2018-02-22

**Authors:** Brice Henry, Valérie Lacroix, Thierry Pirotte, Pierre-Louis Docquier

**Affiliations:** ^1^Service de Chirurgie Orthopédique et Traumatologique, Cliniques Universitaires Saint-Luc, 10 Avenue Hippocrate, 1200 Brussels, Belgium; ^2^Service de Chirurgie Thoracique et Cardiovasculaire, Cliniques Universitaires Saint-Luc, 10 Avenue Hippocrate, 1200 Brussels, Belgium; ^3^Service d'Anesthésiologie, Cliniques Universitaires Saint-Luc, 10 Avenue Hippocrate, 1200 Brussels, Belgium

## Abstract

Minimally invasive procedure for the treatment of pectus excavatum as described by Nuss has been used from 1987. The bar initially introduced blindly is now introduced under thoracoscopic control to increase safety of the procedure. It is usually removed two to three years after its insertion in a one-day procedure. Complications of the bar removal are rare but potentially serious. We report the case of a serious complication which occurred immediately after the Nuss bar removal. A 15-year-old boy underwent a Nuss procedure for a severe pectus excavatum without relevant complication. The bar has been removed two years after its insertion in a minimally invasive procedure. Unfortunately, he developed in the immediate postoperative period a hemopneumothorax due to a right middle lobe laceration which required a middle lobectomy by thoracotomy for hemostasis. Lesions of intrathoracic organs are a rare but potentially serious complication of the removal of the Nuss bar. We now propose to perform this procedure under thoracoscopic control to avoid it. In our experience, adhesions between the bar and the pleura are always present, and those with potential risk for bleeding or inducing intrathoracic organ lesions are suppressed prior to the bar removal.

## 1. Introduction

Minimally invasive treatment of pectus excavatum consisting of the insertion of a metallic bar in the chest was introduced by Nuss [1] in 1987. Initially, the bar was introduced blindly, and thoracoscopy has been later introduced to increase the technique safety. The bar is generally removed between 2 and 4 years after its insertion [2]. Although there are several reports of complications occurring at the time of the bar insertion [3–7], complications related to the bar removal are less known.

Different techniques have been described for the bar removal: Noguchi and Fujita propose to maintain the patient in dorsal decubitus [8], St. Peter et al. propose a method to avoid straightening the bar before removal [9], and Varela et al. propose performing the operation in a patient lying on lateral position [10]. But these techniques remain blind procedures.

Otherwise, life-threatening complications associated with Nuss bar removal exist and are due either to extensive adhesions between the bar and the myocardium or the lungs [11–13] or to bar migration [13–15]. Cases of severe bleeding due to myocardial lesions have been reported during Nuss bar removal [12, 13, 16, 17]. Severe bleeding due to aortic lesions [14] or other intrathoracic lesions have also been reported. Right lobe laceration has been described by Carlucci et al. [18].

We report a new complication of right middle lobe laceration needing lobectomy, and we propose to use thoracoscopy to increase the safety of Nuss bar removal.

## 2. Clinical Case

The patient is a healthy 15-year-old adolescent with severe asymmetrical pectus excavatum. His medical history shows two surgeries for pylorostenosis and for left inguinal hernia. He was occasionally consuming cannabis and alcohol. His height is 164 cm for 52 kg (BMI 19.3 kg/m^2^). Preoperative assessment included a chest CT scan showing a Haller index of 5. He also had a VII-factor deficiency to 19%. A pediatric hematologist concluded that no major hemorrhagic risk was present.

A minimally invasive treatment according to the Nuss technique was decided. The operation was performed under thoracoscopic control. The correction was obtained by using a pectus bar (Biomet®, Jacksonville, FL, USA) fixed to the rib cage with two lateral stabilizers. It was inserted at the maximal location of the deformity which was severe and slightly asymmetrical. Only one pectus bar was used leading to good clinical improvement but a residual deformity. No incident was noted during intervention and particularly no lung injury.

**Figure 1:**
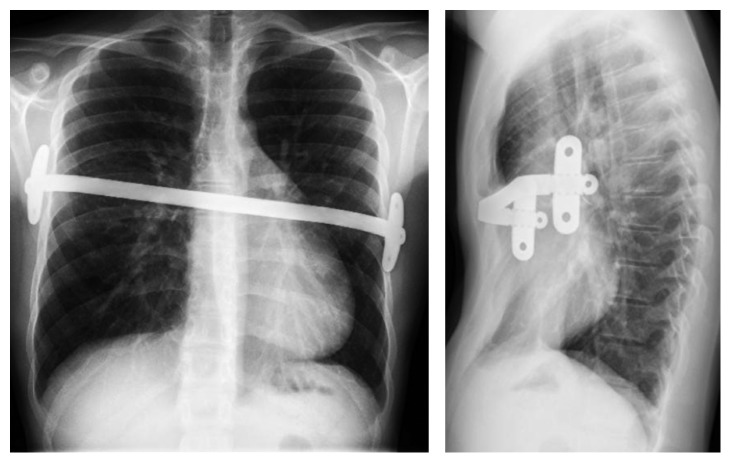
Anteroposterior view and lateral view of thoracic X-ray two years after the insertion of the Nuss bar.

In the postoperative period, he developed pulmonary atelectasis with good evolution with physiotherapy. The thoracic drain was removed the day after surgery. The patient had vomiting with feeding difficulties and therefore stayed in hospital until the 8th postoperative day. The follow-up showed a good evolution despite a slight discomfort at the left stabilization plate (Figure 1).

**Figure 2:**
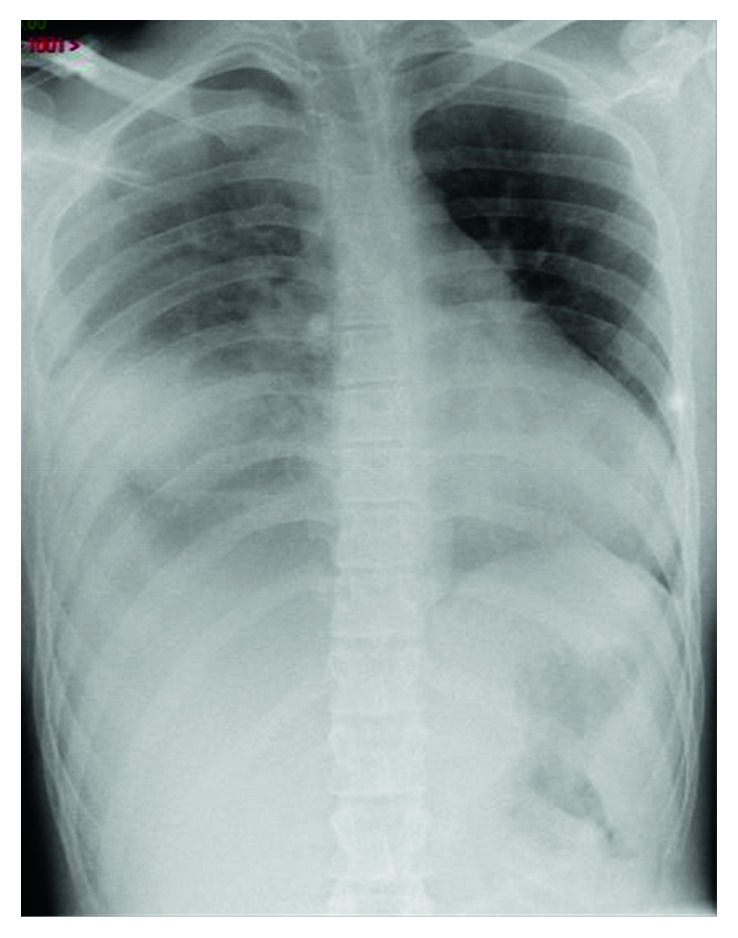
Thoracic radiograph showing the right hemopneumothorax.

After two years, a new assessment of coagulation showed prothrombin time to 1.7 (international normalized ratio) and a stable VII-factor deficiency to 19%. The hemorrhagic risk was not increased, and the bar removal was planned with a daily hospitalization. During the procedure, two incisions were made to remove the left and right stabilization plates. Then, the bar was straightened and removed classically. No perioperative abnormality was observed. In the recovery room, a desaturation began one hour after the end of the surgery. A thoracic wall ultrasonography showed the presence of fluid between the lung and chest wall. A radiograph confirmed a hemopneumothorax (Figure 2).

**Figure 3:**
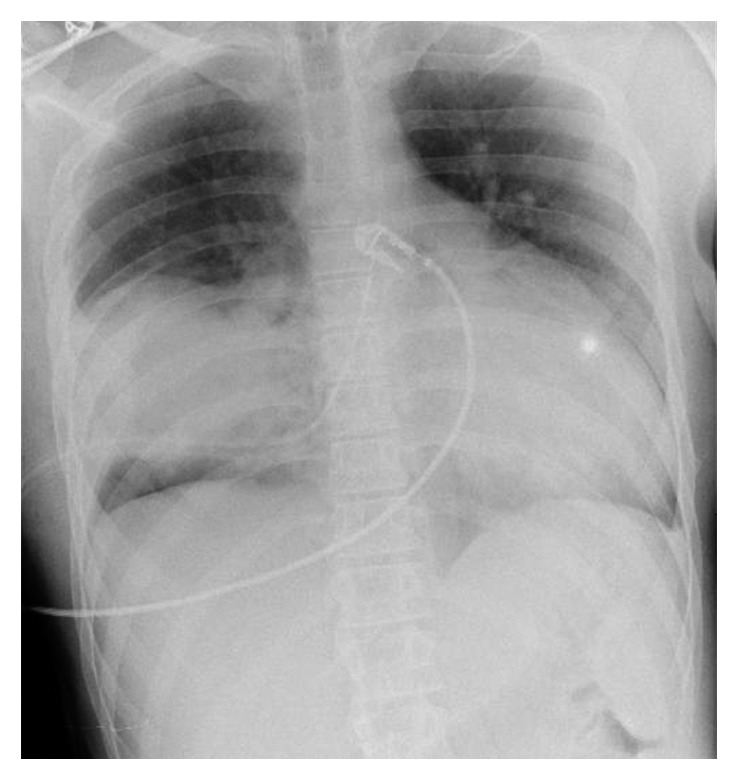
Thoracic radiograph with thoracic drain.

A thoracic drain was inserted which confirmed the hematic nature of the liquid. A second thoracic radiograph showed a decrease of the hemopneumothorax (Figure 3), and desaturation was still present. Hemoptysis occurred.

The thoracic surgeon decided surgical revision by directly performing a right thoracotomy. A right middle lobe laceration was observed. Unfortunately, direct repair of the middle lobe was not possible, and the association to intraparenchymatous hemorrhage needed lobectomy to achieve hemostasis.

The patient evolution was uneventful in the postoperative period. The thoracic drain was removed after two days, and the patient went back to home on the third postoperative day.

## 3. Discussion

Dr. Nuss recommends that the bar should stay in place for 2 to 4 years after its insertion [2]. Thus, the removal of the bar is generally performed two or three years after initial surgery [12, 15, 19]. It is considered a safe procedure and is generally performed as a one-day surgery.

Complications are rare and not well known. In his series of 700 bar removals, Dr. Nuss reports the following complications: 3 pneumothoraxes requiring aspiration and one wound infection [2]. Otherwise, other complications have been described in the literature, with some of which being severe and sometimes life-threatening (Table 1).

Nyboe et al. report a series of 334 cases of Nuss bar removal with a complication rate of 2.4% (8 patients) [19]. Most of them (5 patients) have pneumothoraxes, but 3 have hemothoraxes with one requiring open surgery because of an intercostal artery bleeding. The other two were treated with a chest tube. In a series of 304 Nuss bar removals, Zhang et al. report one intercostal bleeding needing electrocoagulation and one intrathoracic bleeding needing thoracotomy [20]. Molins et al. also reported a case of intercostal artery erosion leading to massive bleeding (1500 cc) and needing extension of incision for hemostasis [21].

Notrica et al. report a case of life-threatening bleeding after removal of a Nuss bar [15]. The patient was treated with two Nuss bars, and X-ray before removal showed anterior migration of the superior bar into the posterior sternal table. The lower bar was removed easily, while removal of the superior bar was harder and followed by bleeding with tachycardia and hypotension. Bleeding stopped with administration of epinephrine, and the thoracotomy did not find a source of hemorrhage. The suspected origin was an internal mammary artery lesion because these vessels were not visible at the site of sternal erosion, and the two arteries were ligated.

Bilgi et al. in their series of 246 Nuss bar removals report a complication rate of 17.5% (*n*=43) [12]. However, these complications included placement of subcutaneous drain (*n*=29), pneumothorax (*n*=3), pleural effusion (*n*=2), chest tube insertion (*n*=6), and major intraoperative bleeding (*n*=3). Major intraoperative bleeding only occurred in cases of removal of double bars, and no thoracotomy was necessary to stop the bleeding. In the first case, thoracoscopy did not show a source of bleeding, and it stopped after bar tract packing. In the other two cases, the bar tract was packed immediately to stop the bleeding, and only one patient needed a chest tube insertion. Thus, they suggest that if major bleeding occurs in a stable patient, immediate packing of the bar tract should be done to stop hemorrhage. They also suggest to pay more attention when removing high bars, turned bars, and bars associated with sternal erosion.

Jemielity et al. reported a case of aortic hemorrhage [14] that occurred during the bar removal (3 years after the Nuss operation). The primary reason for this complication was a rotation of the sternum bar, which caused chronic damage to the aorta and development of an aortomediastinal fistula. Cardiopulmonary bypass and implantation of an aortic prosthesis were required for successful treatment of this complication.

Haecker et al. reported a near-fatal bleeding after transmyocardial ventricle lesion during removal of the pectus bar [16]. The patient was a 20-year-old woman who had undergone thoracotomy and cardiac surgery for transposition of great vessels and mild pulmonary valve stenosis (Mustard technique) at the age of 18 months. Sakakibara et al. also reported a case of massive bleeding during Nuss bar removal due to injury of the heart right ventricle [17]. The patient was a 13-year-old girl who presented with cough for 14 days following Nuss bar insertion. The bar was removed two years later with some resistance while its removal. Massive bleeding occurred in the left thoracic cavity needing sternotomy which showed a 9 cm long perforation of the right ventricle needing repair. Bouchard et al. reported a series of severe cardiac injuries following minimally invasive techniques for repair of pectus excavatum [22]. One case was a lethal cardiac injury at the time of the Nuss bar removal in a 17-year-old boy who underwent a Nuss procedure with revision one month after the first surgery. The patient presented a pericardial effusion which was drained after the revision surgery. The echocardiogram at that time mentioned that the bar did not traverse the pericardium and was also separated from it. Decision to remove the bar was made 6 months after the initial surgery because of pain. The bar was removed without any difficulty, but the patient presented a drop in blood pressure and cardiac arrest leading to a median sternotomy which showed complete obliteration of the space between the heart and the pericardium (“symphysis”). A 2 cm hole in the left ventricle at the place of a suspected adhesion between the bar, the pericardium, and the underlying heart was found. The patient unfortunately died despite heart repair and aggressive resuscitation. Hebra et al. reported another similar case of death at the time of bar removal with autopsy demonstrating ventricular injury and pericardial adhesions [13]. It was noticed that this patient also presented pericarditis needing pericardiocentesis after the first surgery.

Leonhardt et al. described a case of massive arterial hemorrhage at the time of the bar removal needing an emergency sternotomy [11]. The patient was a 14-year-old girl who presented with a recurrence of the chest deformity due to a bar dislocation. A redo surgery was planned 5 months after the first surgery, and bleeding occurred after removal of the bar. Emergency sternotomy showed adhesions between the right lung and the chest wall and an injury of a segment artery of the lower lobe which was ligated. Carlucci et al. [18] described a severe injury occurred after Nuss bar removal. Two years after the Nuss procedure, a bar flipping was noted. The bar was easily removed. A massive right hemothorax occurred: a middle lobe laceration was noted, and lobectomy was performed.

Several techniques are described to remove the Nuss bar. The classical one consists of stabilizing plate removal followed by bar removal by mobilizing the patient to the side.

Noguchi and Fujita reported a technique without need of body position change [8]: benders are inserted subcutaneously into the thoracic wall at both ends of the bar. The bar is then immobilized, straightened, and removed. This technique is also now recommended by Dr. Nuss who states to perform the bar removal with positive pressure ventilation to prevent pneumothorax and recommends to mobilize the both sides of the bar and to unbend it [2]. When straightened, the bar should be removed very slowly under monitoring of vital signs, and a postoperative thoracic X-ray should be performed. St. Peter et al. [9] reported a technique with two operating tables abutted perpendicular to each other forming a T. This allowed preparing the chest circumferentially and wrapping it with two half sheets. By using these techniques, the authors report no complications, but these remain blind techniques. Varela et al. propose a single-incision technique to remove the bar by performing the operation with the patient lying on a lateral decubitus position [10]. Carlucci et al. propose using thoracoscopy to increase safety of bar removal [18] in cases when a cardiac or pulmonary complication occured at the time of bar insertion, if bar displacement is evident or in patients with previous cardiac surgery. However, based on their experience of 20 patients presenting with previous complications at the moment of bar insertion and for whom no complication occurred at the time of blind removal, Bilgi et al. do not suggest the use of an additional procedure for bar removal [23].

Even if it remains rare cases, severe and sometimes life-threatening complications can occur during Nuss bar removal. Adhesions between the intrathoracic organs and chest wall and/or the bar might happen [11–13], displacement of the bar can cause lesions to the great vessels [13, 14], and the bar can also migrate to the chest wall causing sternal erosion [13, 15] or into intrathoracic organs [17]. These are causes of potential severe bleeding at the time of the bar removal. Potential severe bleeding should always be kept in mind when planning Nuss bar removal especially if a prior history of cardiac surgery, a complication associated with the bar insertion (postoperative pericarditis, bar mobility, or displacement), or persistent pain is present [13].

We report here another severe complication occurring after blind removal of the Nuss bar. Since we experienced this complication, we use systematically thoracoscopy to remove Nuss bars. In our experience with this technique, adhesions between the bar and the pleura were always seen. This was not a cause of difficulty for performing the thoracoscopy. Thus, this technique shows, in our minds, several advantages: it allows to suppress adherences potentially responsible of bleeding or intrathoracic organ lesions before removing the bar, direct visualization of the bar progression is useful to detect any potential problem while removing it, and finally, the use of thoracoscopy allows to ensure that no lesion is present after bar removal and before ending the operation.

## Figures and Tables

**Table 1:** Complications after Nuss bar removal identified in the literature. The two cases of death were due to heart lesions.

	Nuss	Nyboe	Zhang	Molins	Notrica	Bilgi	Jemielity	Haecker	Sakakibara	Bouchard	Hebra	Leonhardt	Carlucci	Total
Wound infection	1													1
Pleural effusion						2								2
Pneumothorax	3	5				3								11
Hemorrhagic complications														17
Aortic hemorrhage							1							1
Heart lesion								1	1	1	1			4
Intercostal artery lesion			1	1										2
Sternal erosion					1									1
Lung injury												1	1	2
Hemothorax		3	1											4
Bleeding on the bar tract						2								2
Bleeding without identifiable source						1								1
Death										1	1			2
Others						35								35

*Note.* In the 35 complications reported by Bilgi et al. and named here as “others,” they considered subcutaneous drain insertions (*n*=29) as a complication, and they also reported 6 chest tube insertions without mentioning any reason for that.
